# Cellulosimicrobium cellulans endocarditis: a challenge for detection and treatment in interdisciplinary teams

**DOI:** 10.1007/s15010-025-02551-7

**Published:** 2025-05-19

**Authors:** Tonina T Mueller, Julius Steffen, Clemens Scherer, Matthias WA Angstwurm

**Affiliations:** 1 Department of Medicine I, LMU University Hospital, LMU Munich, Munich, Germany; 2 Department of Medicine IV, LMU University Hospital, LMU Munich, Munich, Germany

**Keywords:** Subacute endocarditis, *Cellulosimicrobium cellulans*, Device therapy

## Abstract

We present a case of subacute endocarditis with the detection of C*ellulosimicrobium cellulans* after tricuspid valve replacement and pacemaker extraction without detection in blood cultures before pacemaker extraction despite multiple testing. This atypical pathogen has rarely been reported in cases of subacute endocarditis, particularly in non-immunocompromised patients. Treatment was initiated using fosfomycin and flucloxacillin and switched to vancomycin upon pathogen detection after pacemaker lead removal and tricuspid valve replacement. Interdisciplinary cooperation between cardiologists, cardiothoracic surgeons, and the infectious disease team enabled an accurate diagnosis and optimized antibiotic therapy before surgical intervention.

## Case report

*Cellulosimicrobium cellulans*, a Gram-positive bacterium, mainly found in soil and wastewater, is an uncommon pathogen in human infection and has only rarely been reported in the literature. Infective endocarditis with *Cellulosimicrobium cellulans* is mostly described in patients with intracardiac foreign material or in immunocompromised patients [1–3].

A 36 year-old patient, who had previously been diagnosed with non-compaction cardiomyopathy and chronic hepatitis B infection without detectable virus load under tenofovir therapy, presented to the department of infectious disease with recurrent fever of unknown origin. Previously, a pacemaker has been implantated in 2004 under the right pectoral muscle and was upgraded to a cardiac resynchronization therapy (CRT) device due to reduced ejection fraction in 2017 implanted on the left side. Prior to recent aggregate replacement surgery in June 2023, the patient tested positive for SARS-CoV-2 infection followed by prolonged fever, which led to an antibiotic treatment with amoxicillin/clavulanic acid for 7 days assuming pulmonary bacterial superinfection.

**Fig. 1:**
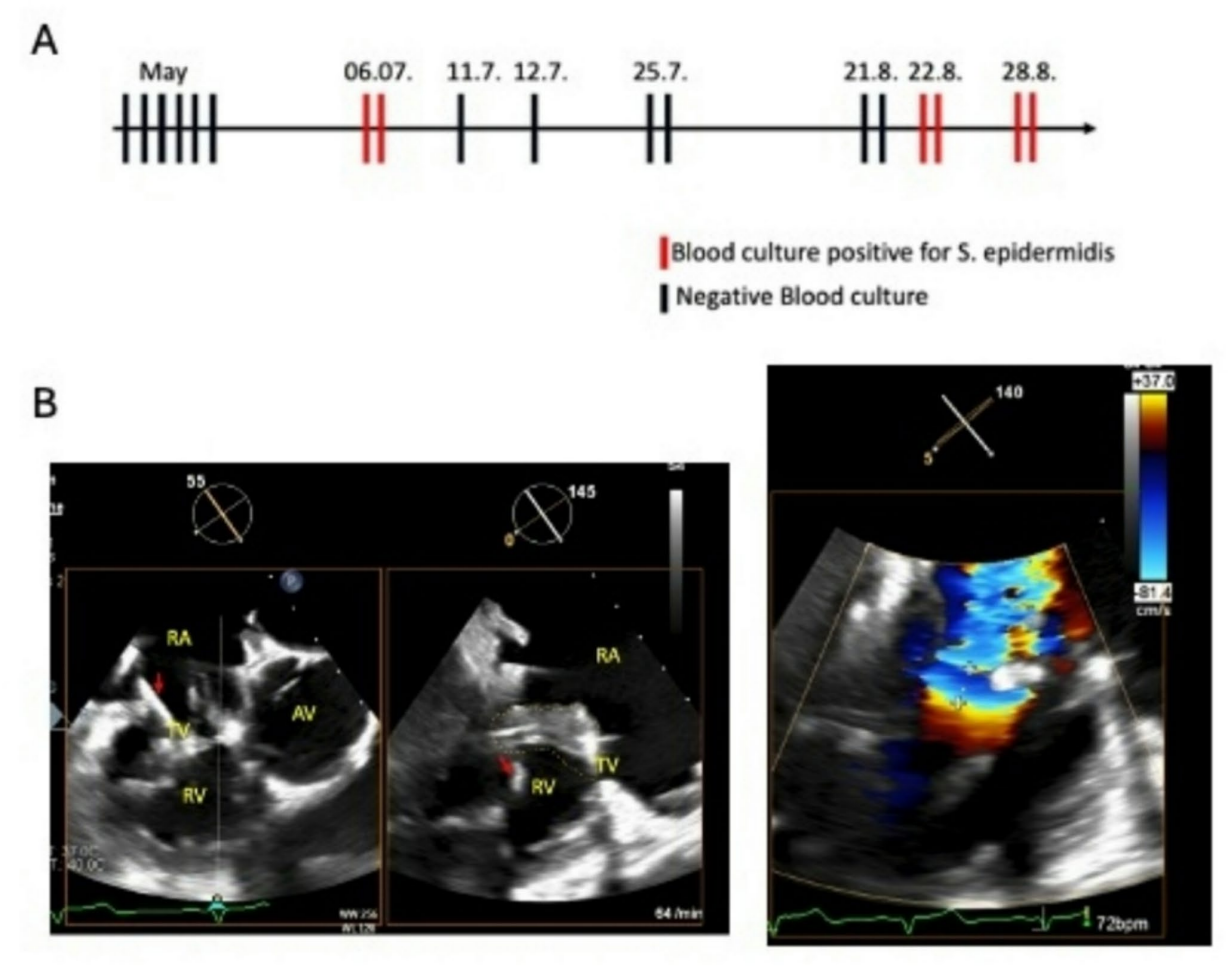
**A**) Blood Culture results until hospital admission. Each line represents one pair of aerobic and anerobic cultures. **B**) Transesophageal echocardiography indicating endocarditic vegetations along the tricuspid valve and CRT leads (right, red arrow) followed by tricuspid regurgitation (right). RA: right atrium, RV: right ventricle, AV: aortic valve, TV: tricuspid valve

**Fig. 2:**
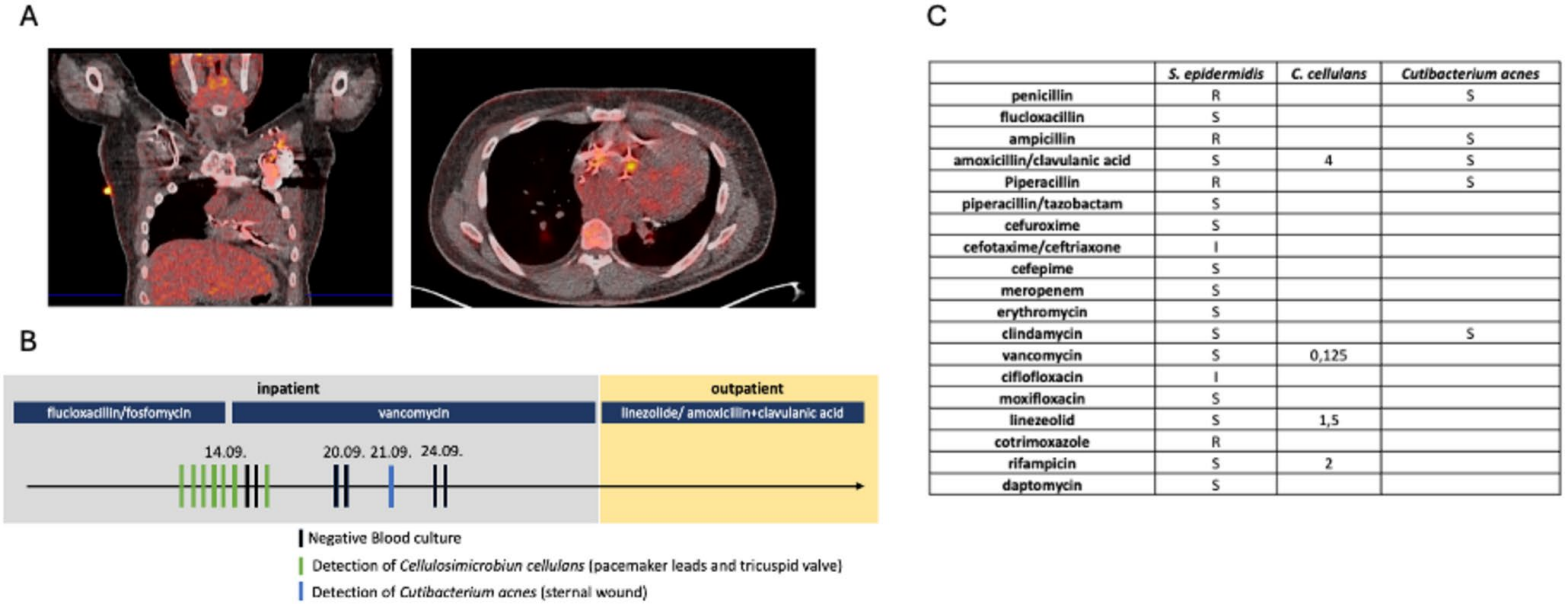
**A**) FDG-PET-CT Scan showing increased metabolic activity along the pacemaker leads and around the aggregate. **B**) Microbiological test results and antibiotic regimen in an inpatient and outpatient setting after hospital admission. **C**) Susceptibility testing for all isolated pathogens (R: resistant; S: sensitive; minimum inhibitory concentration (MIC))

He presented to the infectious disease department with intermittent fever with increasing frequency since the CRT upgrade in 2017 and now worsening after the last aggregate replacement in the previous month due to battery failure. Perioperative antibiotic prophylaxis using cefuroxime had been administered for aggregate replacement. Laboratory testing revealed elevated inflammatory markers (C-reactive protein 5 mg/dl). A thoracic and abdominal computed tomography (CT) scan showed no signs of infection or malignancy. Transthoracic echocardiography was performed without evidence for vegetations along the wires or valves. Peripheral blood cultures were taken and later revealed *Staphylococcus epidermidis*, confirmed in a second testing, prompting admission to a stationary ward for further investigation (Fig. 1A). Intravenous antibiotic therapy comprising flucloxacillin and fosfomycin was initiated immediately after hospitalization. Transesophageal echocardiography (TEE) was performed for endocarditis screening confirming the presence of endocardial vegetations along the ventricular lead and tricuspid valve and suspected abscess formation at the inferior free circumferential region of the tricuspid valve annulus (Fig. 1B). Cardiac CT and whole-body positron emission tomography (PET)-CT scans were performed due to suspected abscess formation, indicating an increased metabolic activity along the pacemaker leads as well as the device (Fig. 2A).

Following 16 days of continuous intravenous antibiotic treatment, an interdisciplinary consensus was reached among cardiologists, cardiothoracic surgeons, and the infectious disease team to proceed with cardiac surgery due to increasing tricuspid valve regurgitation. Surgical exploration revealed severe tricuspid valve destruction precluding valve reconstruction. Abscess formation could not be confirmed. The intervention involved mechanical tricuspid valve replacement, removal of all leads (totaling 5), and extraction of the pacemaker device.

Microbial analysis of the removed leads, pacemaker pocket and tricuspid valve identified *Cellulosimicrobium cellulans*, a pathogen not previously detected in blood cultures, prompting a shift to vancomycin following resistance testing on day 20 (Fig. 2C). Subsequent blood and urine cultures yielded negative results. Post-surgery, *Cutibacterium acnes* was identified in the sternal wound by anaerobe culture. Due to his reduced left ventricular ejection fraction, postoperative cardiocirculatory support therapy with milrinone and iloprost was required.

After another 10 days of continuous vancomycin treatment, a CRT pacemaker system was implanted using epicardial leads. The patient was successfully extubated shortly after and gradually weaned off the catecholamines. Following three weeks of intravenous vancomycin therapy, interdisciplinary discussions led to a change of antimicrobial treatment to an outpatient regimen, consisting of oral linezolid and amoxicillin/clavulanic acid for an additional duration of three weeks (Fig. 2B) totaling six weeks of antibiotic therapy after the implantation of foreign material and since immunosuppression could not be fully excluded in this patient. This resulted in the complete remission of the patient, with the absence of any observable symptoms following the termination of antibiotic therapy.

## Discussion

Subacute endocarditis refers to a form of infective endocarditis, characterized by a gradual onset of symptoms and a more protracted course compared to acute endocarditis, often hampering early diagnosis [4]. The occurrence of *Cellulosimicrobium cellulans* in endocarditis is rare, making it a challenging differential diagnosis and treatment [5]. Infective endocarditis with *Cellulosimicrobium cellulans* associated with subacute infections mostly susceptible for treatment with vancomycin [1–3].

Additionally, in contrast to other cases published in the literature, no positive blood cultures containing *Cellulosimicrobium cellulans* could be obtained prior to pacemaker extraction despite extensive testing. In retrospect, the positive results in blood cultures for *Staphylococcus epidermidis* are likely to be considered contaminations. Nonetheless, the detection of these positive blood cultures and persistent clinical symptoms prompted the initiation of additional diagnostic measures such as TEE or PET-CT scans, emphasizing the importance of obtaining at least two sets of blood cultures. Current guidelines emphasize the early implementation of PET-CT as a diagnostic in suspected endocarditis [6–8]. The severe destruction of the tricuspid valve supports the hypothesis of subacute form of endocarditis consistent with the symptomatic worsening of the patient over the months.

The collaborative approach facilitated comprehensive microbial analysis, enabling the medical team to tailor the antibiotic regimen to the specific pathogen’s characteristics and susceptibility [9]. Furthermore, step-down of the antibiotic therapy to an oral regime allows earlier patient discharge in compliant patients, demonstrating similar efficacy in stable conditions after clearance of bacteriaemia [10]. At one-year follow-up post-discharge, the patient continues to demonstrate good health, with no reported symptoms such as fever.

## Data Availability

No datasets were generated or analysed during the current study.
